# Early sensing of *Yersinia pestis* airway infection by bone marrow cells

**DOI:** 10.3389/fcimb.2012.00143

**Published:** 2012-11-26

**Authors:** Yaron Vagima, Yinon Levy, David Gur, Avital Tidhar, Moshe Aftalion, Hagar Abramovich, Eran Zahavy, Ayelet Zauberman, Yehuda Flashner, Avigdor Shafferman, Emanuelle Mamroud

**Affiliations:** ^1^Department of Biochemistry and Molecular Genetics, Israel Institute for Biological ResearchNess Ziona, Israel; ^2^Department of Infectious Diseases, Israel Institute for Biological ResearchNess Ziona, Israel

**Keywords:** *Y. pestis*, plague, infection, neutrophils, bone marrow, CXCR4, SDF-1, HSPC

## Abstract

Bacterial infection of the lungs triggers a swift innate immune response that involves the production of cytokines and chemokines that promote recruitment of immune cells from the bone marrow (BM) into the infected tissue and limit the ability of the pathogen to replicate. Recent *in vivo* studies of pneumonic plague in animal models indicate that the pulmonary pro-inflammatory response to airway infection with *Yersinia pestis* is substantially delayed in comparison to other pathogens. Consequently, uncontrolled proliferation of the pathogen in the lungs is observed, followed by dissemination to internal organs and death. While the lack of an adequate early immune response in the lung is well described, the response of BM-derived cells is poorly understood. In this study, we show that intranasal (i.n.) infection of mice with a fully virulent *Y. pestis* strain is sensed early by the BM compartment, resulting in a reduction in CXCR4 levels on BM neutrophils and their subsequent release into the blood 12 hours (h) post infection. In addition, increased levels of BM-derived hematopoietic stem and progenitor cells (HSPC) were detected in the blood early after infection. Mobilization of both immature and mature cells was accompanied by the reduction of BM SDF-1 (CXCL-12) levels and the reciprocal elevation of SDF-1 in the blood 24 h post infection. RT-PCR analysis of RNA collected from total BM cells revealed an early induction of myeloid-associated genes, suggesting a prompt commitment to myeloid lineage differentiation. These findings indicate that lung infection by *Y. pestis* is sensed by BM cells early after infection, although bacterial colonization of the BM occurs at late disease stages, and point on a potential cross-talk between the lung and the BM at early stages of pneumonic plague.

## Introduction

The respiratory system is continuously exposed to a variety of invading microorganisms. To limit the ability of pathogens to replicate in the lung, a swift innate immune response is induced following pulmonary infection. This response involves the production of cytokines and chemokines, such as tumor necrosis factor (TNF), interleukin-1 (IL-1), IL-6, IL-8, and interferons (IFNs), and the vigorous recruitment of immune cells from the bone marrow (BM) into the infected tissue (Lukacs et al., 1999; Nathan, 2002). Neutrophils are key players in this response, as has been demonstrated by their involvement in protection against several lung bacterial pathogens including *Pseudomonas aeruginosa* (Tsai et al., 2000), *Legionella pneumophila* (Tateda et al., 2001), *Klebsiella pneumonia* (Matsuzaki and Umemura, 2007), and *Yersinia pestis* (Cowan et al., 2005; Lukaszewski et al., 2005; Spinner et al., 2008, 2010; Laws et al., 2010; O'Loughlin et al., 2010; Eisele et al., 2011). Under steady state conditions, neutrophils are generated and stored in the BM, and only a small fraction (less than 2%) are found in the circulation (Semerad et al., 2002; Raffaghello et al., 2008). In response to infection, neutrophils as well as other hematopoietic cells may be rapidly mobilized from the BM, resulting in a dramatic rise in circulating cell numbers within hours after infection (Rogers and Unanue, 1993; Mantovani et al., 2011; Sadik et al., 2011). Therefore, early sensing of infection and rapid trafficking of neutrophils from the BM to sites of inflammation represent critical steps in the ability of the host to effectively clear the infection.

The mechanism underlying the mobilization process is yet to be fully understood. Recent observations suggest that the chemokine stromal derived factor-1 (SDF-1/CXCL-12) plays an important role in controlling neutrophils mobilization from the BM to the circulation through interaction with its receptor CXCR4 (Suratt et al., 2004; Eash et al., 2009; Delano et al., 2011). CXCR4 is expressed by mature neutrophils as well as immature hematopoietic stem and progenitor cells (HSPC), whereas SDF-1 is constitutively expressed in the BM by various stromal cells (Nagasawa et al., 1996; Kucia et al., 2004; Dar et al., 2005; Guo et al., 2005). Modulation of this chemokine-receptor axis during inflammation leads to a decrease in the amount of surface CXCR4 or BM SDF-1 expression, an event that triggers the release of BM neutrophils (Eash et al., 2009; Delano et al., 2011) as well as HSPCs into the blood (Winkler and Levesque, 2006).

*Yersinia pestis* (*Y. pestis*) is the etiological agent of plague, which has caused millions of deaths in three world pandemics and is still a public health concern in some regions of the world (Pollitzer, 1954; Perry and Fetherston, 1997; Kool, 2005). Primary pneumonic plague results from the inhalation of *Y. pestis* droplets or aerosols, leading to a rapidly progressing disease and high mortality rates in untreated patients, who can spread the disease from person to person (Pollitzer, 1954; Kool, 2005). These characteristics led to the recognition of *Y. pestis* as a potential threat agent (Inglesby et al., 2000). Recent *in vivo* studies in animal models of primary pneumonic plague have indicated that there is an initial delay in the pro-inflammatory response to airway infection with *Y. pestis* (Lathem et al., 2005; Bubeck et al., 2007; Agar et al., 2008; Smiley, 2008). An observed delay in the recruitment of neutrophils to the lung of *Y. pestis*-infected mice was correlated with limited up-regulation of multiple inflammatory cytokines and chemokines. Furthermore, it was recently shown that pulmonary infection with *Y. pestis* also creates a permissive environment for the proliferation of other avirulent bacterial species (Price et al., 2012).

While the lack of an adequate early immune response in the lung during pneumonic plague is well described, the response of distal hematopoietic organs in general and the BM in particular has yet to be fully elucidated. In this study, we analyzed the response of BM cells to pulmonary infection with *Y. pestis* using a mouse model and unrevealed an early systemic immunologic response indicate a potential cross-talk between the lung and the BM at early stages of pneumonic plague.

## Materials and methods

### *Y. pestis* strains and culture conditions

The fully virulent *Y. pestis* Kimberley53 (Kim53) strain was grown on Brain Heart Infusion agar (BHIA, Difco) for 48 hours (h) at 28°C. For intranasal (i.n.) infection, bacterial colonies were harvested and diluted in Heart Infusion Broth (HIB, Difco) supplemented with 0.2% (+) Xylose and 2.5 mM CaCl_2_ (Sigma-Aldrich) to an OD_660_ of 0.01 and grown for 22 h at 28°C in a shaker (100 rpm) as previously described (Tidhar et al., 2009; Zauberman et al., 2009). At the end of the incubation period, the cultures were washed, diluted in saline solution to the required infection dose and quantified by counting colony forming units (cfu) after plating and incubating on BHI agar plates (48 h at 28°C).

### Infection of mice

All of the experiments were performed in accordance with Israeli law and were approved by the Ethics Committee for animal experiments at the Israel Institute for Biological Research. C57BL/6 female mice were purchased from Harlan Laboratories (Israel) and maintained under defined flora conditions at the Israel Institute for Biological Research animal facilities.

I.n. infections were performed as described previously (Tidhar et al., 2009; Zauberman et al., 2009). Briefly, the *Y. pestis* Kim53 strain was grown at 28°C as described above. Cultures were washed and diluted in saline solution to the required infection dose of 110,000 cfu. Prior to infection, mice were anaesthetized with a mixture of 0.5% ketamine HCl and 0.1% xylazine and then infected intranasally with 35 μl/mouse of bacterial suspension. Bacteria were quantified by counting cfu after plating and incubating on BHIA plates as described above. The i.n. LD_50_ of the Kim53 strain under these conditions is 1100 cfu. LD_50_ values were calculated according to the method described by Reed and Muench (1938). All mice infected with 100 LD_50_ of Kim53 pre-grown at 28°C succumbed to the infection within 3–4 days.

### Monitoring bacterial dissemination

To examine bacterial dissemination to the blood and the BM, mice were anesthetized, and blood was collected by cardiac aspiration in heparinized tubes. BM (femurs and tibias) was flushed with a syringe containing 1 ml of cold PBS supplemented with a protease inhibitor mixture (Sigma-Aldrich). Bacterial quantification in BM (cfu/4 bones) or in blood (cfu/1 ml blood) was carried out as described above.

### Flow cytometry analysis

Total BM and blood leukocytes were counted prior to the application of RBCs lysis buffer (Sigma-Aldrich) to the blood samples. Cells were then fixed in 4% paraformaldehyde in PBS for 1 h at room temperature and washed twice in FACS buffer. Levels of Lineage-/Sca-1+/c-Kit+ (LSK) were determined by staining with a mixture of the following Abs: the FITC-conjugated anti-mouse lineage markers CD11b (clone M1/70), CD49b (clone DX5), CD4 (clone GK1.5), CD45R/B220 (clone RA3-6B2), Ly6G (Gr-1) (clone RB6-8C5), CD8a (clone 53-6.7) (Biolegend); PE-conjugated anti-mouse Ly-6A/E (Sca-1) (clone D7) (Biolegend); and APC-conjugated anti-mouse CD117 (c-Kit) (clone 2B8) (Biolegend). Neutrophils (CD11b^+^/Gr-1^high^) were stained with PerCP-Cy5.5-conjugated anti-mouse CD11b (clone M1/70) (eBioscience) and APC-conjugated anti-mouse Ly6G (Gr-1) (clone RB6-8C5) (eBioscience). CXCR4 was stained with PE-conjugated anti-mouse CD184 (clone 2B11) (eBioscience). Cells were stained using standard protocols and appropriate matched isotype control antibodies. The analysis was performed on a FACSCalibur flow cytometer with CellQuest pro (BD Bioscience).

### RT-PCR and quantitative PCR analysis

Total RNA was extracted using Tri-reagent (Sigma-Aldrich) according to the manufacturer's instructions. Two micrograms of total RNA were reverse-transcribed using Moloney murine leukemia virus reverse transcriptase and oligo-dT primers (Promega). Quantitative PCR analysis was performed using an ABI 7500 machine (Applied Biosystems) with SYBR green PCR master mix (Applied Biosystems). The fold change in gene transcript quantity compared with hypoxanthine phosphoribosyl transferase (HPRT) was measured using the comparative (−2^ΔΔCt^) method. Forty cycles of PCR were performed in duplicate for each primer (Table 1).

**Table 1:** **Sequences of primers used in this study**.

**Mouse gene**	**Forward 5′-3′**	**Reverse 5′-3′**
SDF-1 NM_013655	CCCTGCCGGTTCTTCGA	TCAGCCGTGCAACAATCTGA
C/EBPα NM_007678	CGCAAGAGCCGAGATAAAGC	AGGCAGCTGGCGGAAGAT
PU.1 NM_011355	TACAGGCGTGCAAAATGGAA	GACATGGTGTGCGGAGAAATC
FOG1 NM_009569	CAGAGCCTTATCCCCTGAGAGA	TGACAAGGCGCACATATAGCA
G-CSF NM_009971	CCTGGAGCAAGTGAGGAA	AGAGAGTGGCCCAGCAAC
IL-7R NM_008372	AGGCTCCCTCTGACCTGAAAG	CTCTGTGGGATTGTTGTTCTTGTG
FLT3L NM_013520	GTCACTGTGGCCGTCAATCTT	TGGACGAATCGCAGACATTC
PAX5 NM_008782	GGACAGGACATGGAGGAGTGA	CGGCTTGATGCTTCCTGTCT
Rag1 NM_009019	AGCAAGGTAGCTTAGCCAACATG	CTTCGGGTGCCTTTTCAAAG
Ikaros NM_001025597	ATCGAGGCATGGCCAGTAAT	TGCCTCCAACTCCTGACAAAG
HPRT NM_013556	GCAGTACAGCCCCAAAATGG	GGTCCTTTTCACCAGCAAGCT

### Cytokines analysis

Blood and BM extracts were collected and centrifuged at 260 g for 10 min, and the supernatants were collected and stored at −80°C. Before analysis, samples were centrifuged again at 13,000 g for 5 min. SDF-1 levels in BM supernatants were measured by enzyme-linked immunosorbent assay (ELISA) according to the manufacturer's protocol (R&D Systems). Plasma samples were pooled (5 mice per group), and SDF-1 levels were measured using a RayBio mouse cytokine antibody array according to the manufacturer's protocol (RayBiotech, Inc.).

### Histological analysis

Paraffin-embedded femur tissue sections were stained with hematoxylin and eosin alcohol solutions (H&E). Stained bone sections were visualized by light microscopy (A1 Axioscope, Zeiss) and documented by digital photography (AxioCam ICc3 operated by AxioVision Rel. 4.8, Zeiss).

### Detection of F1 and LcrV soluble antigens in BM supernatants

The quantification of F1 and LcrV soluble antigens is based on coupling time-resolved-fluorescence (TRF) of lanthanide to the formation of immune “sandwich” complexes. The assay is performed in a 96 well plate (Nunc maxisorp 442404), in which wells are pre-coated with the relevant capture antibodies (anti-F1 or anti-LcrV). The preparation of the specific anti-F1 and anti-LcrV antibodies was described previously (Flashner et al., 2010). Following the washing and blocking steps, samples were added (50 μL, duplicates) and incubated for 30 min at 37°C. After a washing step, biotinylated-specific antibodies (biotin-anti-F1 or biotin-anti-LcrV) were added in 50 μL assay buffer (Perkin Elmer #4002-0010), and the mixtures were further incubated for 30 min at 37°C. Following the addition of the streptavidin Eu^III^-N1 complex (50 μL, Perkin Elmer #1244-360) and incubation for 15 min at 37°C, the enhancer solution (50 μL, Perkin Elmer #1244-105) was added for the TRF reading. TRF readings were performed using a TECAN Infinite F200 reader under the following conditions: excitation filter: λ = 340 (±35) nm, emission filter: λ = 612 (±10) nm, lag time = 300 μs, integration time = 400 μs, and gain at 150. Naïve mice BM supernatants were used to evaluate the limit of detection (LOD) and the limit of quantification (LOQ) of the assay. LOD = the average of 6 naïve mice sera TRF values + three times the standard deviation, and LOQ = average 6 naïve mice sera TRF values + five times the standard deviation. A calibration curve of spiked antigen (F1 and LcrV) in buffer solution was plotted, allowing the determination of the actual antigen concentration (ng/ml) in the samples.

### Statistical analysis

Differences were analyzed by the two-tailed Student's *t*-test in Excel 2007. All data are presented as the mean plus or minus standard error mean (SEM).

## Results

### Neutrophil mobilization from the BM to the blood is observed at early stages of pneumonic plague

**Figure 1:**
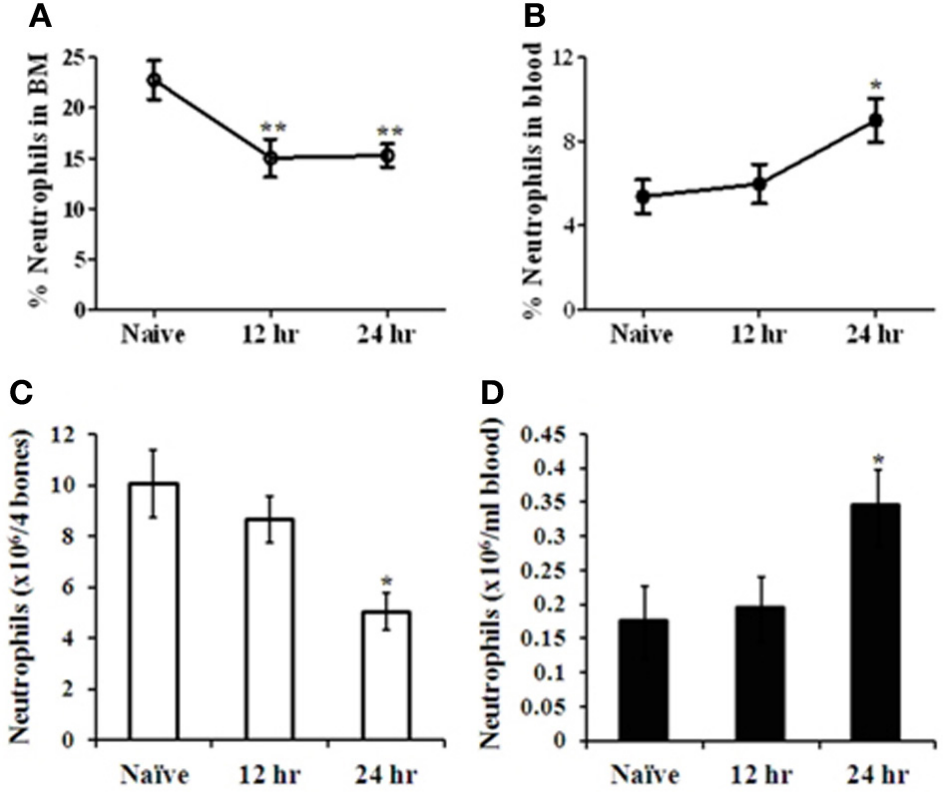
**Analysis of neutrophil mobilization from the BM to the blood at early stages of pneumonic plague**. C57BL/6 mice were infected i.n. with 100 LD_50_ (100,000 cfu) of the virulent *Y. pestis* strain Kim53. The percentage of neutrophils (Gr-1^high^/CD11b^+^) in the BM **(A)** and the blood **(B)** was quantified by flow cytometry. The absolute number of neutrophils was calculated per 4 bones **(C)** and per 1 ml blood **(D)** at the indicated time points post i.n. infection. *n* = 8–12 mice per group. ^*^p < 0.05; ^**^p < 0.01 compared with naïve mice.

The mobilization of mature neutrophils from their storage site in the BM to the blood following infection with *Y. pestis* was evaluated. C57BL/6 mice were infected intranasally with 100 LD_50_ of Kim53, and neutrophil levels were determined at different time points thereafter. FACS analysis of BM and blood-derived cells revealed reciprocal changes in the levels of CD11b^+^/Gr-1^high^ neutrophils by 12–24 h post infection. A significant reduction in the percentage and absolute number of neutrophils was observed in the BM (Figures 1A,C accordingly), followed by an increase in neutrophil numbers in the blood (Figures 1B,D accordingly). These findings suggest that infection of the lung by *Y. pestis* is sensed by BM cells shortly after the infection, leading to the rapid induction of the innate immune response in the BM.

### *Y. pestis* and its soluble F1 and LcrV antigens are detected in the BM only at late stages of pneumonic plague

**Figure 2:**
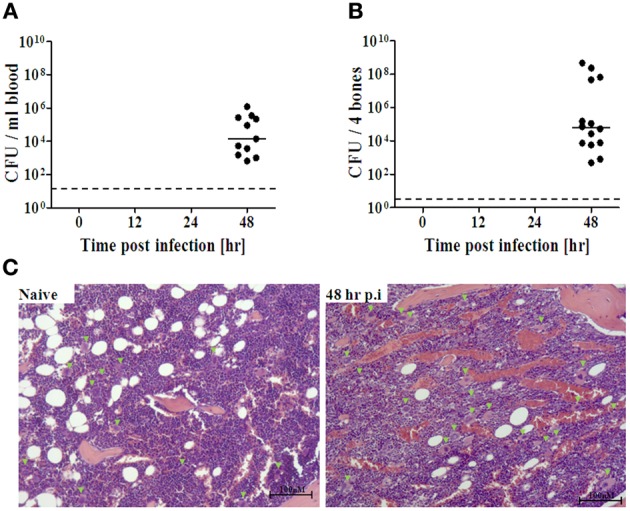
**Quantification of *Y. pestis* dissemination to the BM during disease progression**. Following i.n. infection with Kim53 as described, BM extracts **(A)** and peripheral blood **(B)** were collected at the indicated time points, and the presence of live bacteria was determined by plating serial dilutions of the samples in PBS on BHIA for 48 h at 28°C. The dashed line indicates the limit of detection. The solid line indicates the average of cfu detected. **(C)** Representative figures showing H&E staining of femoral bone in naïve and infected mice at 48 h post infection (original magnification X10). Green arrowheads point to megakaryocytes. Bar = 100 μm.

We next investigated whether the early response of BM cells to distal lung infection may have reflected direct interaction of BM cells with the pathogen within the BM compartment, rather than remote sensing of the infection. Live *Y. pestis* bacteria were detected in the BM only at late stages of disease progression, i.e., 48 h post infection (Figure 2B), concomitantly with the propagation of the pathogen in peripheral blood (Figure 2A). These findings rule out the possibility that rapid sensing of the infection results from direct interaction between the pathogen and BM cells.

Early BM immune responses may also arise from recognition of pathogen-related soluble antigens by BM cells. We therefore measured the level of F1 and LcrV antigens in BM supernatants obtained from Kim53-infected mice. Both assays were validated for the detection of concentrations as low as 0.1 ng/ml (derived from 4 bones). Low levels of the soluble antigens were measured in the BM at 48 h post infection. At this late stage of disease progression, live bacteria were already present in the BM at levels of 10^2^–10^5^ cfu/4 bones (Table 2).

**Table 2:** **Determination of soluble LcrV and F1 proteins in BM of mice in a model of pneumonic plague**.

**Time post infection^a^ (h)**	**Mouse**	**Bacterial load in BM^b^**	**Level of antigen in BM^b^ (average ± SD)**	
		**cfu/4 bones**	**F1 (ng/ml)**	**LcrV (ng/ml)**
	1			
	2			
	3			
24	4	<5	<0.1	<0.1
	5			
	6			
	7			
	1	5 × 10^2^	0.10 (±0.02)	<0.1
	2	8 × 10^3^	0.20 (±0.03)	0.10 (±0.02)
	3	8 × 10^2^	<0.1	0.10 (±0.02)
48	4	1 × 10^5^	2.0 (±0.3)	0.11 (±0.02)
	5	7 × 10^3^	0.20 (±0.03)	<0.1
	6	1.5 × 10^5^	5.0 (±0.7)	0.10 (±0.02)
	7	6 × 10^3^	0.20 (±0.03)	0.10 (±0.02)

a Mice were infected intranasally with 1.1 × 10^5^ cfu/mouse of Y. pestis Kimberley53 virulent strain (100LD_50_).

b BM (femurs and tibias) from infected mice was flushed with 1 ml of PBS (see “Materials and Methods”).

Because limited information is available on the histopathology of the BM during pneumonic plague, we further characterized the BM response during pneumonic plague by performing a histopathology analysis of bone sections obtained from infected mice (Figure 2C). No major differences were observed between tissues from naïve and infected mice during early stages of disease progression (12–24 h post infection, data not shown). However, at late stages of the disease, we observed substantial accumulation of blood vessels along the marrow, visualized as “red patches” resembling BM edema. In addition, increased levels of megakaryocytes were evident in the BM at 48 h post infection.

### The canonical CXCR4-SDF-1 axis is involved in early mobilization of neutrophils from the BM during pneumonic plague

**Figure 3:**
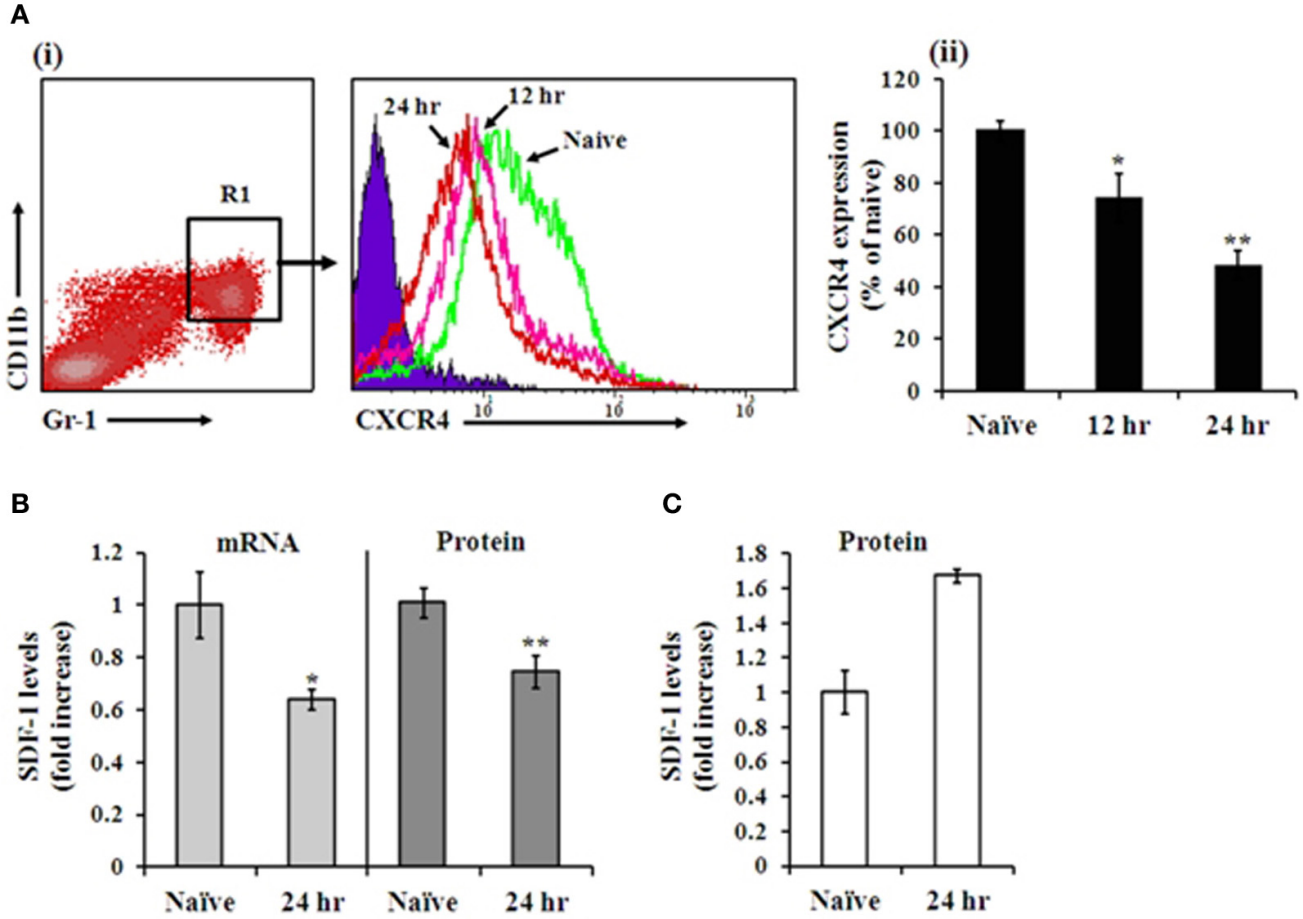
**Analysis of CXCR4 and SDF-1 levels in the BM during pneumonic plague**. **A(i)** Representative FACS histogram analysis showing CXCR4 expression on Gr-1^high^/CD11b^+^ neutrophils at 12 h (pink line) and 24 h (red line) post infection, compared to naïve mice (green line) (blue area indicates isotype control staining). **A(ii)** Summary of CXCR4 expression on Gr-1^high^/CD11b^+^ neutrophils shown in **A(i)**. *n* = 12 mice per group. SDF-1 mRNA levels of total BM cells (**B** left panel), protein concentrations in BM fluids (**B** right panel) and plasma levels **(C)** at 24 h post infection compared with naïve mice. *n* = 8–14 mice per group. ^*^p < 0.05; ^**^p < 0.01 compared with naïve mice.

The CXCR4-SDF-1 axis has been implicated in the mobilization of mature neutrophils and immature HSPC from the BM, in both steady state and alarm situations (Suratt et al., 2004; Dar et al., 2005; Winkler and Levesque, 2006; Eash et al., 2009). To assess the possible involvement of the CXCR4-SDF-1 axis during i.n. infection with Kim53, we quantified the levels of CXCR4 on BM neutrophils and the levels of SDF-1 in the BM and blood during infection. Gated CD11b^+^/Gr-1^high^ neutrophils (R1) exhibited a significant reduction in CXCR4 levels at 12 and 24 h post infection (Figure 3A). In addition, SDF-1 mRNA and protein levels in the BM were reduced at 24 h post infection (Figure 3B), while SDF-1 levels in the blood were elevated (Figure 3C). Taken together, these results imply modulation of the CXCR4-SDF-1 axis during pneumonic plague and are consistent with the establishment of a BM—blood gradient of SDF-1, facilitating neutrophil mobilization early after infection.

### Increased levels of HSPC (LSK) are observed in the blood shortly after airway infection

**Figure 4:**
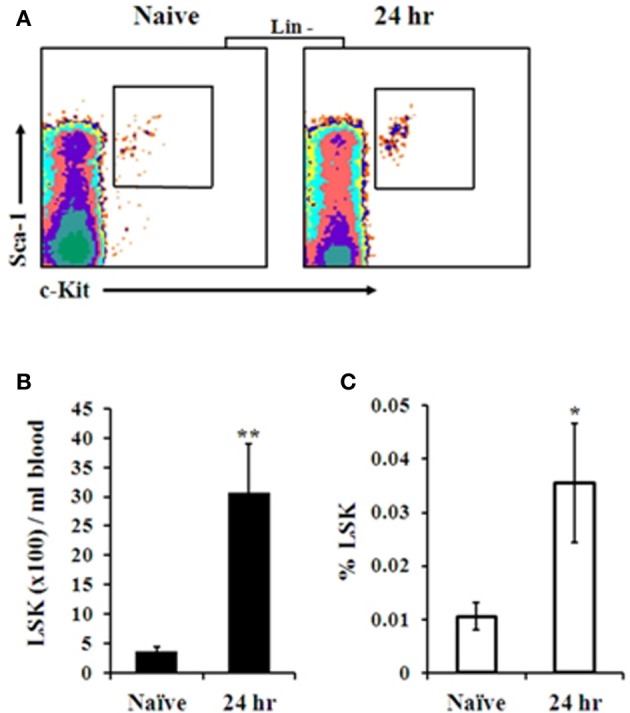
**FACS analysis of HSPC levels in the blood following i.n. infection with *Y. pestis*. (A)** Representative FACS plot of gated LSK cells in the blood of naïve vs. infected mice at 24 h post infection. The absolute number **(B)** and percentage **(C)** of primitive LSK cells per 1 ml blood of naïve vs. infected mice (24 h post infection) are presented. *n* = 8–10 mice per group. ^*^p < 0.05; ^**^p < 0.01 compared with naïve mice.

SDF-1 has been shown to strongly attract human and murine HSPC, controlling their egress and homing from and to the BM under stress conditions (Peled et al., 1999; Hattori et al., 2001; Kollet et al., 2001; Suratt et al., 2004; Dar et al., 2005; Winkler and Levesque, 2006; Eash et al., 2009). The observation that a local gradient of BM-blood SDF-1 is formed shortly after i.n. infection with Kim53 led us to evaluate the level of HSPC mobilization as an additional indication of early sensing of the infection by BM cells. Indeed, elevation of lineage marker-, Sca-1^+^, cKit^+^ (LSK) cell numbers (Figure 4B), as well as their percentage (Figure 4C), was observed in the blood circulation at 24 h post infection with Kim53. These results further support the findings that the BM compartment senses and rapidly responds to *Y. pestis* lung infection.

### Pulmonary infection by *Y. pestis* leads to rapid up-regulation of myeloid genes in BM-derived cells

**Figure 5:**
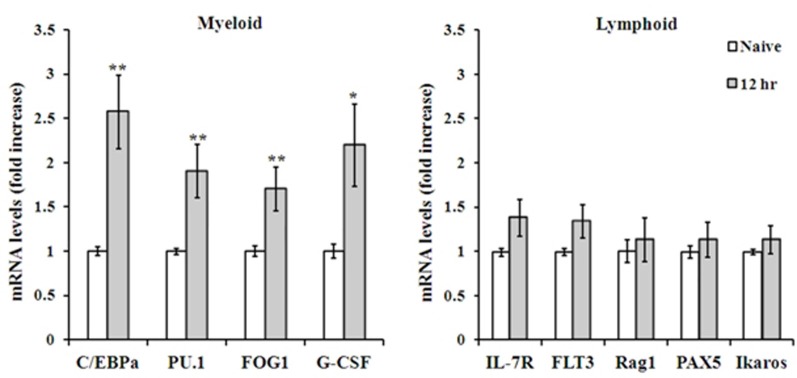
**RT-PCR analysis of myeloid vs. lymphoid associated genes expressed by total BM cells**. Expression levels of representative genes involved in myeloid (left) and lymphoid (right) lineage differentiation, as determined by quantitative PCR of total BM cells mRNA, 12 h post i.n. infection with Kim53. ^*^p < 0.05; ^**^p < 0.01 compared with naïve mice.

Along with the release of hematopoietic precursors into the blood in response to infection, the BM enhances hematopoietic stem cell (HSC) fate decisions in terms of differentiation and maturation into terminally differentiated cells, and a tendency to shift toward granulocyte production has been reported (Ueda et al., 2005; Zhang et al., 2008). To assess whether this tightly controlled process is also modified early after *Y. pestis* i.n. infection, we measured mRNA expression levels of several established factors involved in myeloid and lymphoid differentiation in BM cells from Kim53-infected mice. Transcription levels of the myeloid-associated genes C/EBPα, PU.1, FOG1, and G-CSF were increased in total BM cells by 2–3-fold at 12 h post infection (Figure 5 left panel), while transcription levels of the lymphoid-associated genes IL7-R, Flt3, Rag1, PAX5, and Ikaros did not change at this time point (Figure 5 right panel). These observations further indicate that a rapid and extensive innate immune response is initiated in the BM following i.n. infection with *Y. pestis*.

## Discussion

Innate immune cells are engaged in combating invading pathogens during the first few days after infection, prior to activation of a specific immune response. As the major site of hematopoiesis, the BM has an important role in the early innate immune response and supplies a range of immune cells, neutrophils in particular, that are recruited quickly to inflamed tissues, limiting the expansion and dissemination of the pathogen.

In this study, we assessed the early response of BM cells to pulmonary infection with a fully virulent *Y. pestis* strain by monitoring the release of neutrophils and HSPC from the BM into the blood and evaluating lineage markers of BM cells for further production of myeloid cells, which are required for clearance of the pathogen.

Our findings indicate that an early response is initiated by BM cells after i.n. infection with Kim53, as demonstrated by the rapid mobilization of neutrophils from the BM to the blood within 12–24 h post infection (Figure 1). Moreover, we observed that neutrophil mobilization into the blood was associated with the modulation of the CXCR4-SDF-1 axis. A significant decrease was measured in the levels of CXCR4 on BM neutrophils at 24 h post infection, accompanied with a significant reduction in SDF-1 levels in the BM as well as elevation of SDF-1 levels in the blood circulation at 24 h post infection (Figure 3). The CXCR4-SDF-1 axis is fundamental for neutrophil retention and egress from the BM to the blood (Suratt et al., 2004; Eash et al., 2009; Delano et al., 2011). Various studies imply that neutrophil mobilization from the BM to peripheral organs in general, and the lung in particular during bacterial infections is also CXCR4-SDF-1-dependent (Petty et al., 2007; Delano et al., 2011; Yamada et al., 2011).

Further support for the rapid response of BM cells to *Y. pestis* pulmonary infection came from monitoring HSPC levels in the blood following *Y. pestis* infection. Previous studies have shown that disruption of CXCR4-SDF-1 interaction in the BM and an increase in SDF-1 levels in the peripheral blood, are associated with mobilization of immature HSPC from the BM into the blood (Hattori et al., 2001; Sweeney et al., 2002; Levesque et al., 2003; Dar et al., 2011). HSPC are rare multipotent cells that are capable of generating all the cells of the blood and the immune system under homeostatic conditions and in response to infection (Chandra et al., 2008; Orford and Scadden, 2008; Welner et al., 2008; Zhang et al., 2008). In addition, circulating HSPC may act as patrolling sentinels of infection and modulate the immune response by secreting cytokines, chemokines, and growth factors (Majka et al., 2001; Allakhverdi et al., 2009). Analysis of HSPC numbers and percentages in the peripheral blood following *Y. pestis* i.n. infection revealed a significant elevation at 24 h post infection (Figure 4), further supporting the notion that an early response was induced by BM cells.

**Figure 6:**
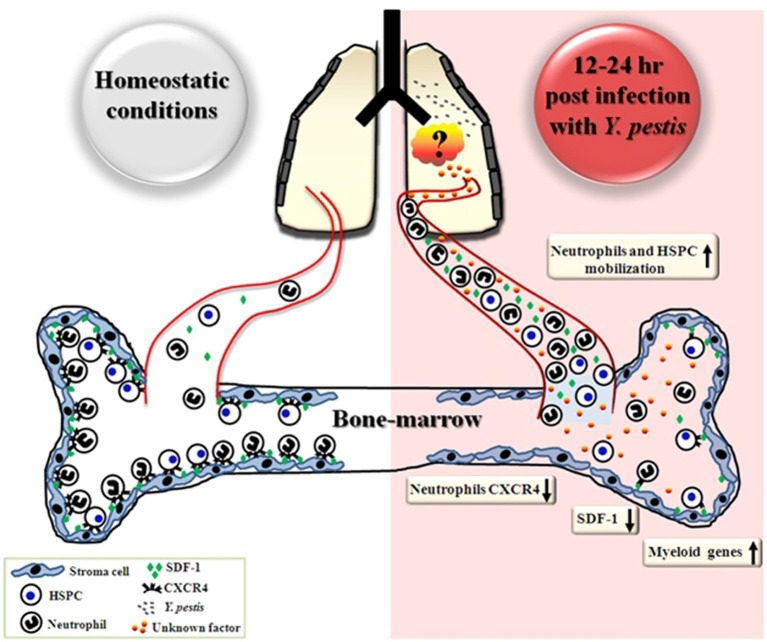
**Schematic representation describing early events in the BM following airway infection with *Y. pestis.*** Early after i.n. infection with *Y. pestis* (12–24 h), reductions in SDF-1 levels in the BM and CXCR4 levels on BM neutrophils are followed by neutrophil mobilization to the blood circulation, where SDF-1 levels are increased. Substantial mobilization of HSPC from the BM to the blood is also observed 24 h after infection. Concurrently, the gene expression profile of myeloid associated genes is up-regulated in total BM cells. During the initial stages of disease progression, live bacteria were not detected in the BM, suggesting that sensing of the infection is indirect and may result from interaction of BM cells with pulmonary-derived factors.

In several experimental models of bacterial infection, the release of various immune cells from the BM following infection, was associated with differentiation toward the myeloid lineage (Shahbazian et al., 2004; Rodriguez et al., 2009). A similar commitment toward the myeloid lineage was rapidly initiated in the BM in response to i.n. infection with Kim53, as demonstrated by the up-regulation of the myeloid-associated genes C/EBPα, PU.1, FOG1, and G-CSF as early as 12 h post infection (Figure 4 left panel). This up-regulation was exclusive to the myeloid lineage, as genes associated with the development of the lymphoid lineage did not exhibit any change in their expression profile (Figure 4 left panel). Taken together, our findings suggest that pulmonary infection with a fully virulent *Y. pestis* strain is sensed by the distal BM compartment shortly after infection, leading to the induction of a prompt innate immune response 12–24 h post i.n. infection (Figure 6). The rapid response of BM cells to *Y. pestis* pulmonary infection suggests a possible cross-talk between the lung and the BM at early stages of infection. Germline-encoded pattern recognition receptors (PRRs) are responsible for sensing the presence of microorganisms by recognizing components conserved among microbial species, namely pathogen-associated molecular patterns (PAMPs). These components vary from plasma membrane proteins to endolysosome and cytoplasmic nucleic acids (Takeuchi and Akira, 2010). The observation that live *Y. pestis* bacilli were detected in the BM only at late stages of disease (Figure 2, LOD is less than 5 cfu/ 4 bones), together with the inability to detect the presence of the pathogen in BM samples from early stages of disease progression using a sensitive PCR-based assay (data not shown), suggest that early sensing of the infection did not result from direct interaction of BM cells with the pathogen within the BM compartment.

Pathogen-derived soluble antigens may also provide a systemic stimulus for BM cells following a remote infection. The soluble form of the F1 capsular antigen of *Y. pestis* has been detected in various clinical specimens (Williams et al., 1986; Chanteau et al., 2000, 2003; Splettstoesser et al., 2004). Previous *in vitro* studies have shown that F1 can activate murine peritoneal macrophage cells, leading to the expression of various pro-inflammatory cytokines (Sodhi et al., 2004). However, determination of soluble F1 levels in BM supernatants from Kim53-infected mice indicated that the antigen could be detected only at late stages of disease progression (48 h post infection), which parallels its appearance in the blood (Flashner et al., 2010). At this time point, *Y. pestis* bacilli had already colonized the BM (Figure 2 and Table 2).

The soluble form of the LcrV protein was recently detected in the bronchoalveolar lavage fluid and blood of intranasally infected mice (Flashner et al., 2010). LcrV is a multi-functional protein that acts as an essential component of the type III secretion system (Cornelis, 2002; Viboud et al., 2003). The soluble form of LcrV has known immunomodulatory functions (Nakajima et al., 1995; Brubaker, 2003; Heesemann et al., 2006), including the inhibition of neutrophil chemotaxis (Welkos et al., 1998). Analysis of soluble LcrV in BM supernatants indicated that low levels could be found in the BM compartment only at late stages of pneumonic plague progression (Table 2).

Taken together our observations indicate that *Y. pestis*-derived factors were not detected in the BM compartment early after infection. We cannot preclude the possibility that the early response of BM cells to lung infection may result from sensing of pathogen-derived nucleic acid or F1 and LcrV antigens in the BM at levels undetectable by the available assays, or by sensing of other *Y. pestis*—derived factors.

BM cells may also respond to host-related factors derived from the primary infection site. Recently, it was shown that i.n. infection with *Y. pestis* induces the expression of type I IFN in the lung (Patel et al., 2012). Interestingly, sensing of respiratory viral infections by BM cells was documented to be mediated by type I IFNs produced at the lung, enabling the education of BM cells for their future encounter with the virus at the infection site (Hermesh et al., 2010). Further studies are needed to identify soluble factors produced by the lung following airway infection with *Y. pestis* that facilitate the early immune response and the egress of neutrophils from the BM to the blood.

Notably, in our i.n. infection model, the local pro-inflammatory response in the lung of infected mice was delayed during the first 24 h post infection, and infiltration of neutrophils into the lung as well as the induction of pro-inflammatory cytokines was observed only at late stages of disease progression, specifically 48 h post infection (unpublished data). This observation is in line with previous studies that have demonstrated a delayed disease progression following i.n. infection with *Y. pestis* bacteria pre-grown at 37°C (Lathem et al., 2005; Bubeck et al., 2007; Smiley, 2008). The early response to infection by BM cells contrasted with the late homing of neutrophils to the lungs raises the question at what stage of the host response does the pathogen interfere?

Neutrophils recruitment from the blood circulation to sites of local infection is a multistep process that involves the migration of cells along a chemotactic gradient and across epithelia. It is well established that inflammatory cytokines (including TNF-a and IL-1b) and neutrophil attractant CXC chemokines initiate the recruitment of neutrophils into infected tissues by diverse actions, as part of the protective innate host response (Charo and Ransohoff, 2006). For example, in mice infected with the respiratory pathogen *Klebsiella pneumoniae*, the rapid induction of many cytokines, including those important for neutrophil chemotaxis, was accompanied with a rapid influx of neutrophils to the lung (Lawlor et al., 2005; Bubeck et al., 2007). However, following *Y. pestis* airway infection the induction of cytokines and chemokines expression in the lung is delayed (Lathem et al., 2005; Bubeck et al., 2007; Agar et al., 2008). Therefore, it is possible to assume that although BM cells respond promptly to the infection by releasing neutrophils into the blood circulation early after infection as demonstrated in this study, these cells cannot find their way into the infected lung due to the lack of an appropriate chemotactic gradient produced by lung resident cells. Moreover, in the absence of a vigorous pro-inflammatory response in the lung there is no induction of adhesion molecules on the endothelium at the infection site. Hence, neutrophils cannot interact with lung endothelial cells, leave the blood vasculature and enter the infected tissue (Phillipson and Kubes, 2011; Sadik et al., 2011). This may provide another possible explanation for the discrepancy between the increased neutrophil levels in the blood at early stages of pneumonic plague and their poor infiltration to the lung.

To the best of our knowledge, this study documents for the first time detection of a rapid innate immune response of BM cells triggered by *Y. pestis* pulmonary infection. Further study will be carried out to address several issues such as identification of the factors that mediate the early sensing of the pathogen by BM residing neutrophils as well as the stage of the multistep process involved in neutrophil migration from the blood to the lung, in which the pathogen interferes.

### Conflict of interest statement

The authors declare that the research was conducted in the absence of any commercial or financial relationships that could be construed as a potential conflict of interest.
